# Gamma-glutamyl transferase to high-density lipoprotein cholesterol ratio is a more powerful marker than TyG index for predicting metabolic syndrome in patients with type 2 diabetes mellitus

**DOI:** 10.3389/fendo.2023.1248614

**Published:** 2023-10-03

**Authors:** Shijun Gong, Shenglian Gan, YuHua Zhang, HaiFeng Zhou, Quan Zhou

**Affiliations:** ^1^Department of Medicine, Jishou University, Jishou, China; ^2^Department of Endocrinology, The First People’s Hospital of Changde City, Changde, China; ^3^Department of Science and Education, The First People’s Hospital of Changde City, Changde, China

**Keywords:** GGT/HDL-C ratio, TyG index, HOMA-IR, insulin resistance, type 2 diabetes mellitus, metabolic syndrome

## Abstract

**Purpose:**

The prevalence of metabolic syndrome (MetS) is increasing globally and has become a global and national public health problem that cannot be ignored as an independent predictor of cardiovascular events, cancer and all-cause mortality. γ-glutamyl transferase (GGT) and high-density lipoprotein cholesterol (HDL-C) are associated with insulin resistance, dyslipidemia and oxidative stress. This study was designed to explore the relationship and predictive performance between γ-glutamyl transferase high-density lipoprotein cholesterol ratio (GGT/HDL-C) and MetS.

**Methods:**

This was a cross-sectional study. MetS was diagnosed from biochemical and anthropometric data in subjects with T2DM. Multivariate logistic regression was used to analyses the relationship between GGT/HDL-C ratio, TyG index and HOMA-IR and MetS in subjects with T2DM. Receiver operating characteristic (ROC) curve was drawn and the areas under the curve (AUC) were used to assess the ability of these indexes in screening MetS in subjects with T2DM. Statistical differences between the AUC values of these indexes were compared. In addition, we performed subgroup analyses and interactions.

**Results:**

769 (70.55%) patients with T2DM were defined as having MetS. patients with MetS had higher anthropometric values and biochemical indicators compared to those without MetS. Multivariate logistic regression analysis of GGT/HDL-C ratio was an independent risk factor for MetS (Per 1 SD increase, OR = 2.49, 95% CI: 1.51, 4.10). According to ROC curve analysis, the value of GGT/HDL-C ratio in predicting MetS in subjects with T2DM was superior to that of TyG index and HOMA-IR. The best cut-off value for GGT/HDL-C prediction was 19.94.

**Conclusions:**

GGT/HDL-C ratio may be an important predictor of MetS in subjects with T2DM, and its predictive power is stronger than that of TyG index and HOMA-IR. The risk of MetS in subjects with T2DM is increased in the presence of a higher GGT/HDL-C ratio.

## 1 Introduction

Metabolic syndrome (MetS) is a group of chronic metabolic disorders characterized by excess waist circumference, diabetes or impaired glucose regulation, dyslipidemia (hypertriglyceridemia and/or low high-density lipoprotein cholesterolemia), hypertension and insulin resistance (1). MetS can increase the incidence of atherosclerotic cardiovascular disease (ASCVD) and is an independent influence on cardiovascular events and the rate of death from all-cause mortality. With the development of urbanization and improvement in living standards, the prevalence of MetS has steadily increased and become a serious threat to human health (2). In China, the prevalence of MetS increased from 24.2% in 2015 to 31.1% in 2017, with 25.2% of those screened positive for one abnormal MetS component, in which the majority had diabetes or were at high risk of diabetes (1). Therefore, further research is urgently needed to identify MetS in high-risk populations and help clinicians take early action.

With the increased prevalence of diabetes, studies have reported a reciprocal effect between diabetes and MetS (3). Therefore, MetS is common in the population with diabetes. The CCMR-3B study showed that the prevalence of MetS in Chinese patients with type 2 diabetes (T2DM) was 57.4%. Of which 71.7% of diabetic patients were positive for at least one abnormal MetS component other than glucose abnormalities (4). In a UK cohort study, more than two-thirds of people with diabetes were found to have one or more components of the metabolic syndrome such as hyperlipidemia and hypertension (5). Despite the discrepancies in the results of the above studies, which are based on the different population characteristics of the study population. It is undeniable that diabetic patients are at high risk for MetS and therefore it becomes urgent to explore a simpler, easier, and more effective tool to screen diabetic patients for MetS to avoid cardiometabolic complications.

Anthropometric indicators have been a convenient predictor of MetS for a long time (6–8). However, anthropometric indicators are subject to many instabilities and often change after the body has reacted pathologically, whereas biochemical indicators are more likely to change in response to changes in the body size and composition and before the onset of disease. Objective indicators have rarely been explored between MetS.

Insulin resistance (IR) is a major pathophysiological mechanism in MetS and diabetes. Therefore, early and accurate identification of IR is clinically important for implementing prevention strategies and optimizing syndrome management. There are many methods available for estimating insulin resistance, ranging from complex techniques to simple indices. Methods for assessing IR fall into three broad categories, namely dynamic tests, simple indices, and biochemical indices (9, 10). Each method has its own strengths and limitations. High insulin clamp test (HEC), Frequent Sampling Intravenous Glucose Tolerance Test (FSIVGTT) are the gold and silver standards for assessing IR. However, they are expensive, time consuming, invasive and non-physiologic methods (10). Biochemical markers (e.g., fasting insulin, insulin-like growth factor-binding protein-1 (IGFBP-1), sex hormone-binding globulin (SHBG)), while providing useful information on IR status, cannot be interpreted alone and need to be combined with the other tests (9). Relatively simple alternatives have been proposed, such as homeostasis modeling assessment (HOMA-IR). HOMA-IR is the most commonly used indirect indicator to assess IR, however is vulnerable to the accuracy of insulin testing equipment (11). Compared to health systems in high-income countries, health systems in middle- and low-income countries have limited resources for laboratory facilities, which are not available in all health systems, HOMA-IR reproducibility is poor. As widespread measurement of insulin resistance or circulating insulin levels is not feasible in clinical practice, a simpler, accurate, practical and more cost-effective index of assessment is sought in a context of enhanced use of existing resources rather than the introduction of entirely new methods. To address this limitation, researchers have developed the triglyceride-glucose (TyG) index which is considered to be a reliable index for assessing IR (12, 13), and is better than the HOMA-IR (14).

The GGT/HDL-C ratio, a new metric proposed in a recent study, is not only less susceptible to interference, but is also readily available and has been shown to be a good predictor of risk for T2DM, non-alcoholic fatty liver disease (NAFLD) and is strongly associated with IR (15–17). Liver is one of the main target organs for insulin and liver dysfunction is closely associated with the development of MetS (18). Gamma-glutamyl transferase (GGT) is a common marker of liver dysfunction. Numerous studies have demonstrated that GGT is a predictive biomarker for MetS and that the risk of MetS increases with increasing GGT levels (19–21). In addition, The Framingham Heart Study has shown that GGT is positively associated with BMI, blood pressure, triglycerides and glucose (22). High-density lipoprotein cholestero (HDL-C) is a multifunctional structural protein with anti-inflammatory, antioxidant and cholesterol efflux effects, long considered the ‘good’ cholesterol (23). HDL-C stimulates glucose uptake and improves β-cell function (24). Studies have shown that lower HDL-C is associated with IR, dyslipidemia and obesity; and that IR is a potential mechanism for dyslipidemia, characterized by lower HDL-C and increased TG in the MetS component (25).

Given the correlation between GGT and HDL-C and MetS, it is crucial to study the association between GGT/HDL-C ratio and MetS. However, there is still a lack of studies reporting the association between GGT/HDL-C ratio and MetS. The aims of this study (1) were to assess the association between GGT/HDL-C ratio and MetS in subjects with T2DM and its predictive performance, (2) were to compare which of the three indexes, GGT/HDL-C ratio, TyG index and HOMA-IR, could more accurately screening for MetS in subjects with T2DM.

## 2 Materials and methods

### 2.1 Participants

**Figure 1:**
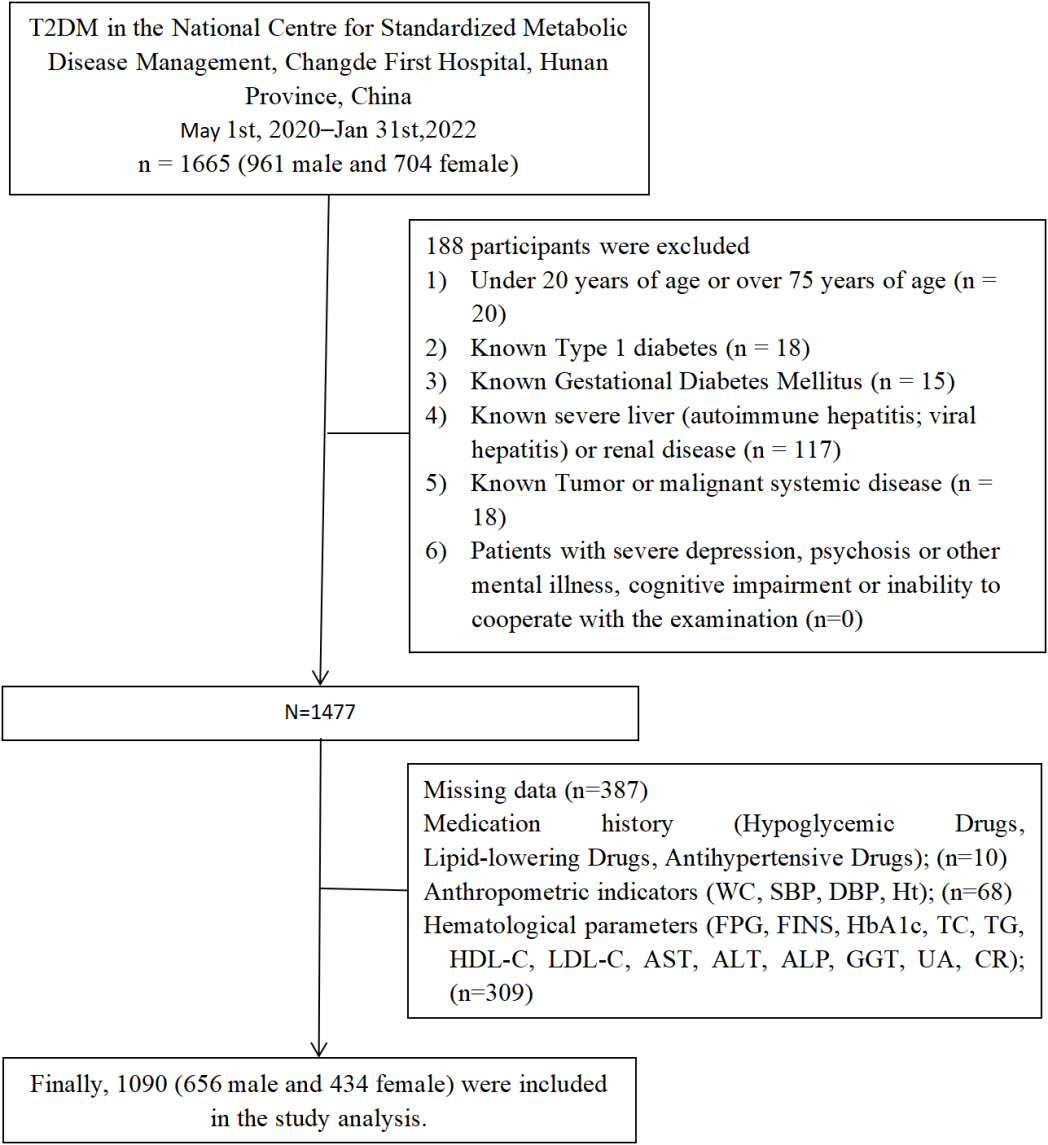
Flowchart of the study population. SBP, systolic blood pressure; DBP, diastolic blood pressure; WC, waist circumstance; HbA1c, Glycated haemoglobin; FPG, fasting plasma glucose; FINS, fasting insulin; ALT, alanine aminotransferase; AST, aspartate aminotransferase; ALP, alkaline phosphatase; GGT, γ-glutamyl transpeptidase; TD, triglyceride; TC, total cholesterol; HDL-C, high-density lipoprotein cholesterol; LDL-C, low-density lipoprotein cholesterol; CR, creatininel UA, uric acid.

The study population was derived from T2DM aged 20 to 75 years who met the WHO (1999 diagnostic criteria for diabetes mellitus) at the National Standardized Metabolic Disease Management Centre (MMC), Changde First People’s Hospital, Hunan Province, China, from May 2020 to January 2022 (n=1665). Exclusion criteria included: (1) Under 20 years of age or over 75 years of age (n=20); (2) patients with type 1 diabetes, gestational diabetes mellitus, diabetes with acute complications (n=33); (3) patients with a known history of liver disease, including cirrhosis, chronic hepatitis and autoimmune hepatitis and renal disease(n=117); (4) patients with major depression, schizophrenia or other psychiatric disorders, cognitive impairment or inability to cooperate with the examination (n=0); (5) patients with malignant neoplasm of the liver system and other tumor diseases (n=18); (7) Those with missing data ((n=387), including through Medication history (n=10); Anthropometric indicators (waist circumference, systolic blood pressure, diastolic blood pressure, height; n=68); Hematological parameters (fasting plasma glucose, fasting insulin, glycated hemoglobin, total cholesterol, triglycerides, low-density lipoprotein, high-density lipoprotein, alanine aminotransferase, alanine aminotransferase, gamma-glutamyl transferase, serum creatinine, serum uric acid; n=309). Finally, 1090 were included in the study analysis. (**Figure 1**).

### 2.2 Data collection

Sociodemographic data such as gender, age, smoking status, alcohol consumption status, medication use (lipid-lowering drugs, antihypertensive drugs, glucose-lowering drugs) and whether women are menopausal were collected through an independent digital patient record system and questionnaires. Smoking status is categorized as non-smoking, ex-smoking and current smoking. Alcohol consumption status is categorized as non-drinking, occasional drinking and daily drinking. Waist circumference (WC) was assessed using a flexible tape measure by placing it horizontally at the middle point between the lower rib and the iliac crest in standing positions. Weight and height measurements required participants to be barefoot and wear light clothing during the measurement. Body mass index (BMI) (kg/m2) was calculated as the weight (kg) divided by the square of the height (m). After resting for 5-10 minutes in a quiet environment, systolic blood pressure (SBP) and diastolic blood pressure (DBP) were measured in the seated position using a standard mercury sphygmomanometer according to a standardized protocol, and three consecutive blood pressure readings were recorded, each at least 5 minutes apart. The complete physical examination is done by a professional endocrinology nurse. All measurements were carried out in strict accordance with national standards.

Fasting venous blood samples collected after 10-12 hours of fasting included alanine aminotransferase (ALT), aspartate aminotransferase (AST), alkaline phosphatase (ALP), gamma-glutamyl transferase (GGT), fasting plasma glucose (FPG), fasting insulin (FIN), glycated hemoglobin (HbA1c), total cholesterol (TC), triglycerides (TG), low-density lipoprotein (LDL-C), high-density lipoprotein (HDL-C) and serum creatinine (CR), serum uric acid (UA), glomerular filtration rate (eGFR). The estimated glomerular filtration rate (eGFR) was calculated by using the modified diet equation in renal disease. eGFR (mL/min/1.73 m2) was calculated using the modified diet in renal disease. eGFR = 186 × Scr (mg/dL) - 1.154 × age - 0.203 × 0.742 (if female) × 1.233 (26). TyG index was calculated as ln [TG (mg/dL) × FPG (mg/dL)/2] (27). HOMA-IR was calculated as [FIN (μIU/mL) × FPG (mmol/L)]/22.5 (28).

### 2.3 Definition

We defined MetS according to the guidelines for preventing and controlling type 2 diabetes in China (2019 Edition) (29). People with three or more of the following are diagnosed with MetS: (1) Abdominal obesity (central obesity): waist circumference ≥ 90 cm for men, ≥ 85 cm for women. (2) Hyperglycemia: fasting blood glucose ≥ 6.1 mmol/L (110mg/dl) or blood glucose ≥ 7.8 mmol/L (140mg/dl) 2 h after glucose load and/or diagnosed with diabetes and treated. (3) Hypertension: blood pressure ≥ 130/85 mmHg and/or hypertension confirmed and treated. (4) Fasting triglyceride (TG) ≥ 1.70 mmol/L (150mg/dl). (5) Fasting high-density lipid cholesterol (HDL-C) < 1.04 mmol/L (40mg/dl).

### 2.4 Statistical analysis

Continuous variables were expressed as mean ± standard deviation or median (quartiles: Q1, Q3), and differences between groups were examined using independent samples t-tests or Mann-Whitney U-tests. Categorical variables were expressed as frequencies (percentages) and chi-square tests or Fisher exact tests were used to test for differences between groups. Pearson’s correlation coefficients were calculated to assess the correlation between GGT/HDL-C ratio, TyG index and HOMA-IR. Multivariate logistic regression was used to analyze the relationship between the three indexes and MetS in subjects with T2DM. A series of sensitivity analyses were performed to ensure the stability of the results. We transformed the target independent variables into quartile-based categorical variables and calculated trend p-values, plotted subject operating characteristic (ROC) curves and calculated area under the curve (AUC) values to assess the predictive power of the three indexes for MetS. The optimal cut-off value for the three predictors of MetS was determined, defined as the point at which the “sensitivity + specificity - 1” value reached its maximum (Jorden’s index). Two-by-two comparisons were made to compare the area under the curve (AUC). In addition, subgroup analyses were conducted including categorical variables such as age, gender, BMI, smoking and alcohol consumption to explore whether statistical differences existed across groups and between MetS. All statistics and analyses were performed by Empower Stats (R) version 4.2 and R software version 3.4.3. Two-sided P values <0.05 were considered statistically significant.

## 3 Results

### 3.1 Prevalence of sub-population of MetS components in subjects with T2DM

**Table 1** shows the gender specific prevalence of MetS and its components in the study population. The overall prevalence of MetS was 70.55%, with a higher prevalence in males (76.37%) than females (61.75%). The results showed that males had a higher prevalence of MetS, increased blood pressure, increased TG, decreased HDL-C, and abdominal obesity. In short, there was a difference in the prevalence of MetS and component abnormalities between the genders.

**Table 1:** Prevalence of MetS and its components by gender.

Sex	Total	MetS		Elevated HP		Elevated TG		Reduced HDL-C		Central obesity	
	n	n	%	n	%	n	%	n	%	n	%
Male	656	501	76.37	337	51.37	412	62.8	283	43.14	481	73.32
Female	434	268	61.75	218	50.23	226	52.07	80	18.43	290	66.82
Total	1090	769	70.55	555	50.92	638	58.53	363	33.3	771	70.73

n, The number of cases with MetS, or abnormal components; %, The prevalence of cases with MetS or abnormal components among male, female, or total subjects. MetS, metabolic syndrome; HP, blood pressure; TG, triglyceride; HDL-C, high-density lipoprotein cholesterol; Central obesity, Waist circumference ≥ 90 cm for male, ≥ 85 cm for female.

### 3.2 Characteristics of the participants

Based on exclusion criteria, this study ultimately included 1090 participants (656 males and 434 females) with a mean age of 52.2 ± 11.1 years. **Table 2** describes the basic characteristics of all participants. Significantly higher levels of SBP, DBP, BMI, WC, smoking, drinking, HbA1c, FPG, FIN, TC, TG, LDL-C, UA, ALT, AST, GGT, TyG index, HOMA-IR, GGT/HDL ratio were observed in participants in the MetS group compared to the non-MetS group. The proportion of participants taking lipid-lowering drugs, antihypertensive drugs, glucose-lowering drugs was also significantly higher in the MetS group. While HDL-C levels were significantly lower than in subjects without MetS.

**Table 2:** Baseline characteristics of the participants.

Parameter	Total	Non-MetS Group	MetS Group	P-value
N	1090	321	769	
Age(years)	51.68 (11.03)	52.15 (10.91)	51.48 (11.08)	0.356
DBP (mmHg)	82.71 (10.85)	77.28 (9.37)	84.98 (10.63)	<0.001
SBP (mmHg)	133.86 (19.22)	127.37 (19.82)	136.57 (18.30)	<0.001
BMI (kg/m2)	25.91 (3.74)	23.13 (2.66)	27.08 (3.50)	<0.001
WC (cm)	92.16 (10.45)	83.52 (7.90)	95.76 (9.20)	<0.001
Course of diabetes(month)	39.00 (4.00-96.00)	46.00 (8.00-86.00)	37.00 (2.00-96.00)	0.787
HbA1c (%)	8.47 (2.27)	8.24 (2.46)	8.57 (2.18)	0.029
FPG (mmol/L)	8.75 (3.37)	8.40 (3.14)	8.90 (3.45)	0.026
FIN (pmol/L)	67.86(42.27-104.09)	44.80 (31.31-70.19)	77.09 (51.65-116.47)	<0.001
ALT (U/L)	25.00 (17.00-39.00)	20.50 (15.00-30.75)	36.39 (29.96)	<0.001
AST (U/L)	23.00 (18.00-29.50)	21.00 (17.00-26.00)	24.00 (19.00-31.00)	<0.001
ALP (U/L)	75.00 (62.00-90.75)	76.00 (63.00-93.00)	75.00 (62.00-90.00)	0.231
GGT(U/L)	28.00 (19.00-47.00)	20.00 (16.00-29.00)	34.00 (23.00-54.00)	<0.001
CR (umol/L)	64.07 (20.86)	59.53 (16.81)	65.97 (22.08)	<0.001
UA(μmol/L)	338.27 (84.57)	304.96 (73.10)	352.26 (85.19)	<0.001
eGFR(mL/min/1.73m2)	118.65 (34.34)	119.58 (99.85-140.05)	113.20 (94.24-135.95)	0.016
TG (mmol/L)	1.90 (1.35-2.86)	1.46 (0.85)	2.28 (1.70-3.31)	<0.001
TC (mmol/L)	4.84 (1.25)	4.78 (1.06)	4.87 (1.33)	0.24
HDL-C (mmol/L)	1.20 (0.32)	1.38 (0.35)	1.13 (0.27)	<0.001
LDL-C (mmol/L)	2.76 (0.90)	2.72 (0.92)	2.77 (0.90)	0.373
TyG index	2.14 (0.81)	1.64 (0.59)	2.35 (0.79)	<0.001
HOMA-IR	3.84 (2.31-7.04)	2.56 (1.68-4.34)	4.62 (2.85-8.10)	<0.001
GGT/HDL-C	25.00 (15.92-44.10)	15.60 (11.04-22.31)	31.87 (20.19-53.00)	<0.001
Sex (n, %)				<0.001
Male	656 (60.18%)	155 (48.29%)	501 (65.15%)	
Female	434 (39.82%)	166 (51.71%)	268 (34.85%)	
Smoking status (n, %)				<0.001
Never	663 (60.83%)	225 (70.09%)	438 (56.96%)	
Former smoker	95 (8.72%)	20 (6.23%)	75 (9.75%)	
Current smoker	332 (30.46%)	76 (23.68%)	256 (33.29%)	
Alcohol status (n, %)				<0.001
Never	722 (66.24%)	248 (77.26%)	474 (61.64%)	
Ever	86 (7.89%)	19 (5.92%)	67 (8.71%)	
Everyday	282 (25.87%)	54 (16.82%)	228 (29.65%)	
Menopausal status (n, %)				0.419
No	320 (73.73%)	126 (75.90%)	194 (72.39%)	
Yes	114 (26.27%)	40 (24.10%)	74 (27.61%)	
Hypoglycemic Drugs (n, %)				0.172
No	146 (13.39%)	50 (15.58%)	96 (12.48%)	
Yes	944 (86.61%)	271 (84.42%)	673 (87.52%)	
Lipid-lowering Drugs (n, %)				<0.001
No	941 (86.40%)	300 (93.46%)	641 (83.44%)	
Yes	149 (13.60%)	21 (6.54%)	121 (16.56%)	
Antihypertensive Drugs (n, %)				<0.001
No	796(73.03%)	289 (90.03%)	507 (65.93%)	
Yes	294(26.97%)	32 (9.97%)	262 (34.07%)	

BMI, body mass index; SBP, systolic blood pressure; DBP, diastolic blood pressure; WC, waist circumstance; HbA1c, Glycated hemoglobin; FPG, fasting plasma glucose; FIN, fasting insulin; ALT, alanine aminotransferase; AST, aspartate aminotransferase; ALP, alkaline phosphatase; GGT, gamma glutamyl transferase; TG, triglyceride; TC, total cholesterol; HDL-C, high-density lipoprotein cholesterol; LDL-C, low-density lipoprotein cholesterol; CR, creatinine; UA, uric acid; eGFR, Estimated glomerular filtration rate; GGT/HDL-C,GGT/HDL-C ratio; TyG index, Triglyceride-glucose index; HOMA-IR, homeostasis model assessment of insulin resistance; MetS, metabolic syndrome.

### 3.3 Pearson’s correlation coefficients were calculated to assess the correlation between GGT/HDL-C ratio, TyG index, and HOMA-IR in subjects with T2DM

**Table 3** showed the Pearson’s correlation between the GGT/HDL-C ratio, TyG index, HOMA-IR in subjects with T2DM. Significant weak positive correlation was found between the GGT/HDL-C ratio and HOMA-IR (r=0.3697, p<0.001), significant moderate-strength positive correlation was found between the GGT/HDL-C ratio and TyG index showed a significant moderately strong positive correlation (r=0.4367, p<0.001),and a similar correlation between TyG index and HOMA-IR (r=0.4675, p<0.001).

**Table 3:** Pearson’s correlation between GGT/HDL-C ratio, TyG index, HOMA-IR in subjects with T2DM.

Variables	GGT/HDL-C	TyG index	HOMA-IR
GGT/HDL-C	1.0000	0.4367*	0.3697*
TyG index	0.4367*	1.0000	0.4675*
HOMA-IR	0.3697*	0.4675*	1.0000

GGT/HDL-C, GGT/HDL-C ratio; TyG index, Triglyceride-glucose index; HOMA-IR, homeostasis model assessment of insulin resistance; MetS, metabolic syndrome. Statistical significance level *P < 0.0001.

### 3.4 Association between GGT/HDL-C ratio, TyG index, HOMA-IR and prevalence of MetS in subjects with T2DM

We used multivariate logistic regression analyses to association between three indexes (GGT/HDL-C ratio, TyG index, HOMA-IR) and MetS in subjects with T2DM. The unadjusted and adjusted model for the 3 groups are shown in the **Table 4**. In this study we converted the GGT/HDL-C ratio to a Z-score for analysis. In model I a positive association was found between GGT/HDL-C ratio and MetS (odd ratio (OR) per increasing standard deviation (SD) 2.01, 95% confidence interval (CI) 1.38, 2.93) and a progressively increasing risk of MetS in quartiles of GGT/HDL-C ratio [OR (Q1: 1, Q2: 2.85, Q3: 4.96, Q4: 8.91, trend p<0.0001). The positive correlations between them remained stable and strengthened in model II (OR for GGT/HDL-C ratio was 3.28 per SD increase, 95% CI OR for TyG index per SD increase was 7.39, 95% CI 5.16, 10.59; OR for HOMA-IR per SD increase was 2.25, 95% CI 1.46, 3.45). In conclusion, the GGT/HDL-C ratio was an independent risk factor for MetS in patients with T2DM.

**Table 4:** Risk of incident MetS in subjects with T2DM according to GGT/HDL-C, TyG index, and HOMA-IR indices.

	Non-adjusted	Model ^I^ OR (95% CI)	Model ^II^ OR (95% CI)
GGT/HDL-C Z-score	5.78 (3.79, 8.81) <0.0001	2.01 (1.38, 2.93) 0.0003	3.28 (1.94, 5.54) <0.0001
GGT/HDL quartile			
Q1	ref	ref	ref
Q2	3.73 (2.62, 5.33) <0.0001	2.85 (1.89, 4.31) <0.0001	3.40 (2.14, 5.38) <0.0001
Q3	8.49 (5.68, 12.71) <0.0001	4.96 (3.10, 7.94) <0.0001	6.49 (3.78, 11.13) <0.0001
Q4	20.12 (12.10, 33.44) <0.0001	8.91 (4.99, 15.91) <0.0001	13.32 (6.46, 27.49) <0.0001
P for trend	<0.0001	<0.0001	<0.0001
TyG-index Z-score	3.70 (3.01, 4.55) <0.0001	3.72 (2.90, 4.77) <0.0001	7.39 (5.16, 10.59) <0.0001
TyG-index quartile			
Q1	ref	ref	ref
Q2	2.81 (1.99, 3.98) <0.0001	3.11 (2.03, 4.76) <0.0001	4.77 (2.91, 7.81) <0.0001
Q3	6.38 (4.32, 9.43) <0.0001	7.45 (4.63, 11.97) <0.0001	16.47 (9.06, 29.93) <0.0001
Q4	20.36 (11.92, 34.79) <0.0001	21.31 (11.43, 39.73) <0.0001	66.64 (28.45, 156.10) <0.0001
P for trend	<0.0001	<0.0001	<0.0001
HOMA-IR Z-score	3.99 (2.78, 5.73) <0.0001	2.06 (1.44, 2.96) <0.0001	2.25 (1.46, 3.45) 0.0002
HOMA-IR quartile			
Q1	ref	ref	ref
Q2	2.27 (1.60, 3.21) <0.0001	1.44 (0.95, 2.16) 0.0824	1.70 (1.09, 2.65) 0.0197
Q3	3.75 (2.59, 5.43) <0.0001	1.86 (1.20, 2.88) 0.0057	2.32 (1.42, 3.79) 0.0008
Q4	8.78 (5.61, 13.74) <0.0001	4.17 (2.48, 7.03) <0.0001	5.25 (2.82, 9.77) <0.0001
P for trend	<0.0001	<0.0001	<0.0001

**^I^** Included variables were age and sex, BMI.

**^II^** Included variables were age and sex; BMI; diabetes course; HbA1c; Lipid-lowering Drugs; Antihypertensive Drugs; Smoking status; Alcohol status; Alanine aminotransferase; Aspartate aminotransferase; Alkaline phosphatase; low-density lipoprotein cholesterol; total cholesterol; creatinine; uric acid; Estimated glomerular filtration rate.

GGT/HDL-C, GGT/HDL-C ratio; TyG index, Triglyceride-glucose index; HOMA-IR, homeostasis model assessment of insulin resistance; MetS, metabolic syndrome; OR, odd ratio; CI, confidence interval.

### 3.5 GGT/HDL-C ratio, TyG index, HOMA-IR predicted MetS in patients with T2DM

**Figure 2:**
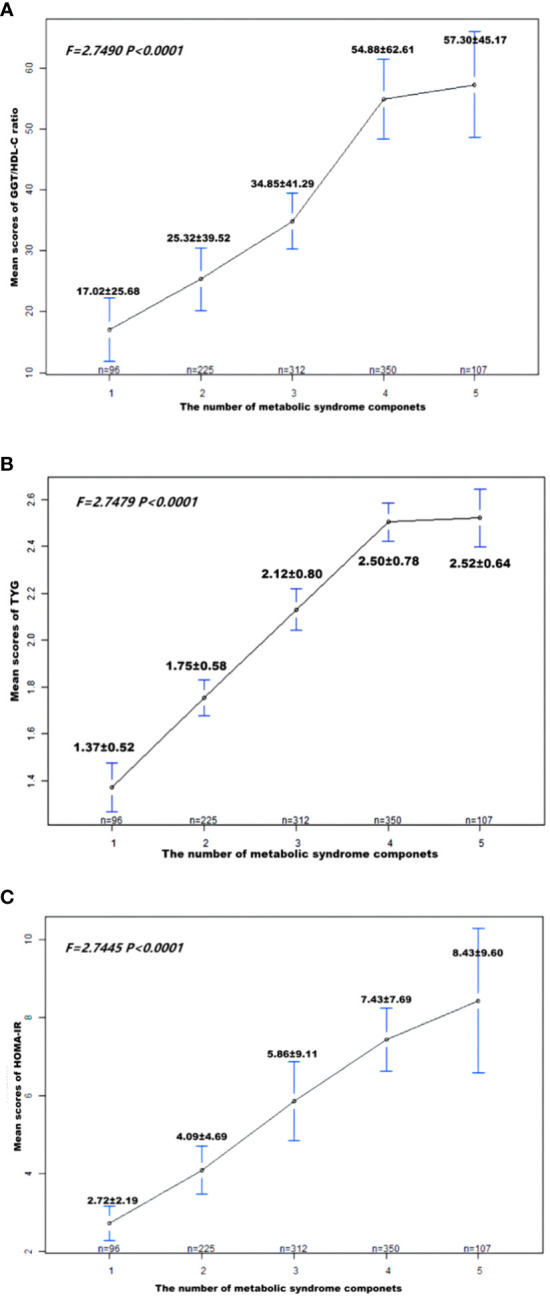
Patient **(A)** GGT/HDL-C ratio; **(B)** TyG; **(C)** HOMA-IR means score and standard error, according to the number of metabolic syndrome components. *P*-values were obtained by Fisher’s exact test. MetS, Metabolic syndrome; TyG, triglyceride glucose; GGT/HDL-C ratio, the ratio of Gamma-glutamyl transferase divided by high-density lipoprotein cholesterol; HOMA-IR, homeostasis Model Assessment of Insulin Resistance.

**Figure 3:**
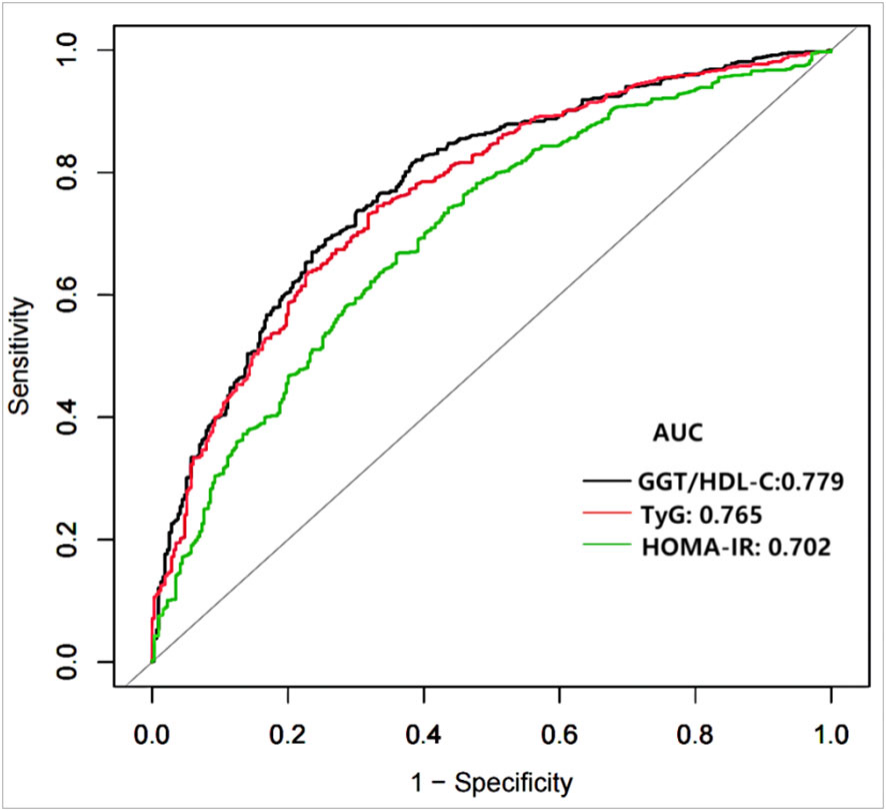
Receiver operating characteristics curves of GGT/HDL-C ratio and other indexes for identifying MetS. MetS, Metabolic syndrome; TyG, triglyceride glucose; GGT/HDL-C ratio, the ratio of Gamma-glutamyl transferase divided by high-density lipoprotein cholesterol; HOMA-IR, homeostasis Model Assessment of Insulin Resistance.

**Figure 2** demonstrates that TyG index, HOMA-IR, and GGT/HDL-C ratios increase in proportion to the number of MetS components in subjects with T2DM. **Table 5** and **Figure 3** show the AUC values of the three indexes used to screen for having MetS in subjects with T2DM. Of the three indicators examined, GGT/HDL-C ratio had the highest AUC of 0.779 (95% CI: 0.749-0.809), with an optimal threshold of 19.94 (sensitivity 0.738, specificity 0.698). TyG index was next with an AUC of 0.765 (95% CI: 0.734-0.795) and an optimal cut-off value of 1.82 (sensitivity 0.745, specificity 0.669). The lowest AUC was found for HOMA-IR with an AUC of 0.702 (95% CI: 0.667-0.736), and an optimal cut-off value of 3.31 (sensitivity 0.667, specificity 0.640). In brief, GGT/HDL-C ratio is a good indicator for screening for MetS in subjects with T2DM, as well as GGT/HDL ratio is stronger than TyG index and HOMA-IR. The study also reported additional details of the three indexes, such as negative predictive value (NPV) and positive predictive value (PPV). We then compared the prevalence of MetS with the escalation of GGT/HDL ratio, TyG index and HOMA-IR. We found that the prevalence of MetS in subjects with T2DM increased as GGT/HDL-C ratio increased (**Supplementary Table 1**). The threshold we obtained was a rather important turning point, beyond which the prevalence of MetS in subjects with T2DM almost doubled. Again, the above results were validated for stability in Bootstrap resampling (number=500) analysis. (**Supplementary Figure 1**)

**Table 5:** ROC analysis of TyG index, HOMA-IR, GGT/HDL-C ratio for predicting MetS in subjects with T2DM.

Test	AUC	95%CI low	95%CI upp	Cutoff Value	Specificity	Sensitivity	NPV	PPV
GGT/HDL-C	0.779	0.749	0.809	19.94	0.698	0.738	0.543	0.845
TyG index	0.765	0.734	0.795	1.82	0.669	0.745	0.539	0.834
HOMA-IR	0.702	0.667	0.736	3.31	0.640	0.667	0.462	0.806

GGT/HDL-C, GGT/HDL-C ratio; TyG index, Triglyceride-glucose index; HOMA-IR, homeostasis model assessment of insulin resistance; MetS, metabolic syndrome; ROC, Receiver-operating-characteristic; AUC, Area under the curve; CI, Confidence interval; NPV, negative predictive value; PPV, positive predictive value.

### 3.6 Comparison of AUC values between GGT/HDL-C ratio, TyG index, and HOMA-IR

**Table 6** shows AUC value differences between the indices used to screen for MetS for TyG inde, HOMA-IR and GGT/HD-C ratio. the difference in AUC values between GGT/HD-C ratio and HOMA-IR was statistically significant (Z=-3.261; p=0.001), similarly that between HOMA-IR and TyG index was statistically significant (Z=-2.856; p=0.004). However, the difference between GGT/HD-C ratio and TyG index was not statistically significant (p=0.591).

**Table 6:** Comparison of AUC values among TyG, HOMA-IR, GGT/HDL-C ratio.

IDI	Z	95%CI low	95%CI upp	P value
GGT/HDL -C vs TyG	-0.537	-0.100	0.057	0.591
GGT/HDL-C vs HOMA-IR	-3.261	-0.206	-0.051	0.001
TyG vs HOMA-IR	-2.856	-0.180	-0.034	0.004

GGT/HDL-C, GGT/HDL-C ratio; TyG index, Triglyceride-glucose index; HOMA-IR, homeostasis model assessment of insulin resistance; MetS, metabolic syndrome; ROC, Receiver-operating-characteristic; AUC, Area under the curve; CI, Confidence interval; IDI, Integrated Discrimination Index.

### 3.7 Subgroup analysis

**Figure 4:**
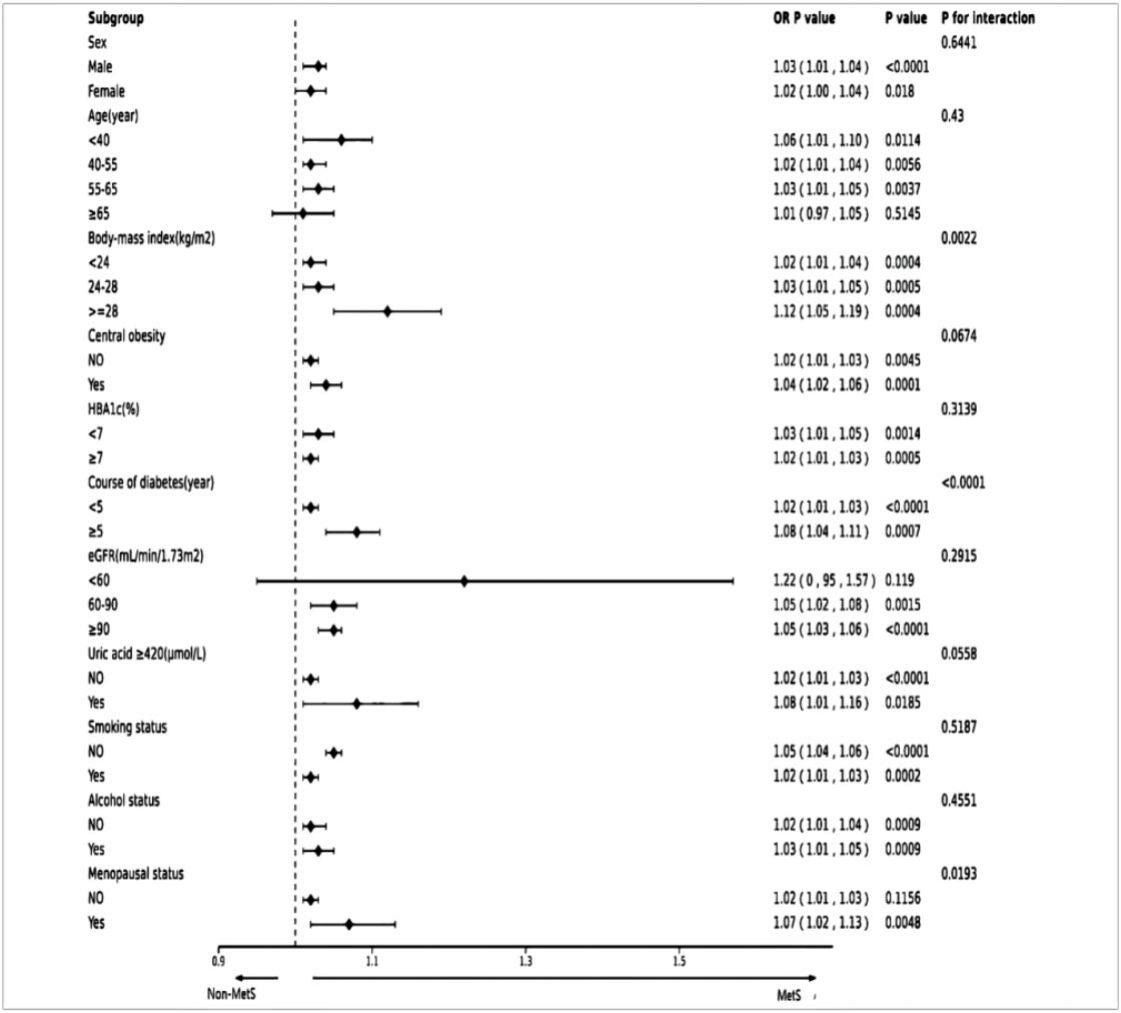
Forest plot of subgroups. The dotted line indicates a hazard ratio equal to 1.0. MetS, Metabolic syndrome.

In subgroup analyses, we further assessed whether the GGT/HDL-C ratio and MetS differed between populations by stratified analysis and interaction tests. As shown in the forest plot (**Figure 4**), the interaction for BMI and duration of diabetes was significant. (p-values for interactions were 0.0022 and <0.0001), whereas tests for interactions for age, gender, HbA1c, smoking, alcohol consumption, abdominal obesity and UA were not statistically significant (p-values for interactions >0.05). Menopausal status was collected in the original data, so we also adjusted for this in women and the results showed an interaction p=0.0193.

## 4 Discussion

In this study, the GGT/HDL-C ratio, TyG index and HOMA-IR were used to determine the optimal cut-off values for screening for MetS in subjects with T2DM. The GGT/HDL-C ratio is a relatively accurate indicator for identifying MetS in subjects with T2DM and has a higher and more discriminatory value than the TyG index and HOMA-IR. Based on the results of the ROC analysis, GGT/HDL-C ratio, TyG index and HOMA-IR cut-off values were 19,94 (AUC=0.779), 1.82 (AUC=0.765) and 3.31 (AUC=0.702) respectively.

IR is an independent risk factor for the development of MetS and diabetes. Therefore, it is important to identify IR in a clinically significant way. Methods of assessing IR fall into three broad categories, namely dynamic tests, simple indices and biochemical indices (9, 10). Among the dynamic tests such as HEC is considered a reference technique. However, HEC has limitations including technical demands, laboriousness, time consumption, expense, and non-physiologic methods. Due to the complexity of HEC, FSIVGTT was preferred as an alternative option. Unlike HEC, FSIVGTT relies on dynamic glucose and insulin data obtained before and after intravenous glucose administration. Nonetheless, it is time-consuming and limited by the availability of appropriate software, and it does not provide results in diabetic subjects (10). In addition, measurement of biochemical markers (e.g., fasting insulin, IGFBP-1, SHBG) can provide useful information about IR status. However, the results of these indices should not be interpreted in isolation and need to be interpreted in the context of other findings, including anthropometric measures such as BMI and WC as well as biochemical measures. Age, sex, and race should also be considered. More importantly, biological variability needs to be considered (9). HOMA-IR is the most used measure of insulin resistance for epidemiological studies. Compared to the high glucose clamp test, HOMA-IR requires only insulin and glucose testing and has the advantage of being less invasive, less costly, and simpler to perform (30, 31). Previous studies have reported that the HOMA-IR index is a good indicator for defining MetS and IR in adults. Research conducted in different countries has reported that the threshold for HOMA-IR to predict adult MetS ranges from 1.70-2.83 (AUC=0.68-0.82) (32, 33). Our study found a higher threshold for HOMA-IR than the above-mentioned studies, based on the fact that we studied T2DM, and our results were similar to those of a diabetic population study in Iran (34). However, there are limitations to this indicator, which is based on fasting glucose and insulin levels, as previously mentioned; one of these is that the measurement of insulin levels is not only expensive. It is also difficult to measure insulin levels in laboratories and health facilities in many developing countries or low-income areas, and there are problems with standardization. On the other hand, diabetic patients receiving insulin therapy or subjects with severely impaired or lost pancreatic beta-cell function can seriously interfere with the measurement results. In contrast, the TyG index and GGT/HDL-C ratio is measured without the need for fasting insulin levels. These tests have the advantage of being able to review lipid parameters and GGT levels, are less expensive than insulin, and can be done by all clinical laboratories.

TyG index is a simple and effective alternative marker for identifying insulin resistance by combining fasting glucose and triglycerides (12, 35). A series of clinical studies have confirmed the close association between TyG index and MetS (27, 33, 36). This study determined that TyG index (AUC=0.765) was a valid marker for diagnosing MetS superior to HOMA-IR (AUC=0.707). The findings are consistent with those of Primo David et al. (TyG index (AUC= 0.746), HOMA-IR (AUC= 0.682)) (37). TyG index is also associated with other diseases. A real-world single-centre study reported that TyG index was more strongly associated with atherosclerosis in T2DM patients compared to HOMA-IR (38). It is important to note that TyG index is susceptible to hyperlipidemia and diabetes as it is a composite index consisting of fasting TG and FPG. In most studies, TG and FPG are only examined at baseline and certain acute illnesses, or unexpected events may lead to stress hyperglycemia and affect TyG index. In addition, dietary habits can greatly affect TG levels. In addition, most studies on TyG index use in MetS have been conducted in middle-aged or older adults, and there is a lack of information on the value of TyG index in younger participants (39).

Multivariate logistic regression analysis showed a strong correlation between GGT/HDL-C ratio and MetS after adjusting for all available confounders. Each 1 SD increase in GGT/HDL-C ratio was associated with a 228% increase in the prevalence of MetS. ROC analysis showed that GGT/HDL-C ratio was an accurate indicator of MetS screening (ACU= 0.779, 95% CI: 0.749-0.809), with an optimal threshold of 19.94, which has a high sensitivity and specificity.

GGT/HDL-C ratio has good predictive performance for the risk of T2DM and NAFLD. Xie, Wangcheng et al. found that GGT/HDL-C ratio is an important predictor of T2DM (15), with an AUC of 0.750 and an optimal threshold of 17.92. It is lower than that of the present study. Feng, Guofang et al. explored the predictive performance of GGT/HDL-C ratio for NAFLD risk (17). They discovered that the GGT/HDL-C ratio had an AUC of 0.799 and an optimal threshold of 21.3, which was slightly higher than that of the present study. Compared with T2DM, GGT/HDL-C ratio had the best predictive performance for NAFLD and the second-best predictive ability for MetS. This is because GGT and HDL-C are more strongly correlated with NAFLD, which is either a symptom and cause of MetS (40). Anyway, GGT and HDL-C are routinely tested in clinical laboratories compared to HOMA-IR and TyG indexes. GGT/HDL-C ratio is indeed a convenient and cost-effective indicator for screening the risk of MetS in subjects with T2DM.

Mechanisms underlying the association between high GGT/HDL-C ratios and MetS remain unclear and are currently thought to be related to insulin resistance, dyslipidemia and oxidative stress in MetS pathology (41, 42). Elevated GGT is a marker of intracellular triglyceride accumulation and is associated with overweight/obesity and insulin resistance, not alcohol consumption (43, 44). In addition to its ability to reflect the involvement of insulin resistance in the development and progression of MetS by representing liver dysfunction and intrahepatic lipid accumulation (41). On the other hand GGT is involved in oxidative stress in organisms through the regulation of glutathione, which is linked to the pathophysiological development of MetS. Some investigators suggest that elevated serum GGT reflects oxidative stress and chronic inflammation, which also contributes to the development of MetS (45, 46). GGT not only positively correlates with numerous inflammatory factors such as C-reactive protein and interleukin-6, but also itself mediates the conversion of leukotriene C4 to leukotriene D4, which enhances the development of inflammation (15). Numerous previous studies have demonstrated that GGT is a useful biomarker for predicting MetS and its components, and that the risk of MetS increases with increasing levels of GGT (22).

Dyslipidemia is a risk factor for MetS, and reduced HDL-C is the most common clinical disorder of lipid metabolism. As part of the diagnosis of MetS, HDL-C can improve cellular uptake of glucose in skeletal muscle cells and insulin secretion from beta cells and helps protect β-cells from apoptosis caused by cholesterol accumulation by promoting cholesterol efflux (15, 16). HDL-C also has anti-inflammatory and antioxidant effects, directly increasing beta cell survival by inhibiting apoptosis caused by oxidized LDL-C and inflammatory factors, HDL-C decreased levels may reflect reduced beta cell function and insulin resistance (23). Study demonstrates an independent negative correlation between HDL-C and IR (24, 47). IR may be an underlying mechanism for dyslipidemia, characterized by increased TG and decreased HDL-C in the MetS component. IR leads to increased release of fatty acids from adipose tissue and increased hepatic TG synthesis, contributing to increased plasma TG levels, hypertriglyceridemia contributes to decreased HDL-C through enhanced clearance of TG-rich HDL by hepatic lipase due to enhanced clearance of TG-rich HDL by hepatic lipase (25).

In the context of a high GGT/HDL-C ratio, both elevated GGT levels and reduced HDL-C levels are associated with an increased risk of MetS. The optimal threshold for the GGT/HDL-C ratio is 19.94, which means that when T2DM patients have a GGT/HDL-C ratio above 19.94, we need to pay extra attention to them as they are at high risk of MetS. Clinicians need to urge them to improve their lifestyle and promote healthy eating and physical activity to help them prevent or delay MetS early.

In addition, the heterogeneity of MetS is reflected in alterations in the transcriptome and associated pathways (48, 49). The anti-aging gene Sirtuin 1, whose involvement in glucose, fatty acid, and cholesterol metabolism has been demonstrated, and is involved in the biological processes of oxidative stress and inflammation by activating the transcriptional activity of downstream genes (50, 51). In recent years, Sirtuin 1 has been shown to be associated with insulin resistance and to be important for glucose-dependent insulin secretion and the protection of pancreatic β-cell mass (52). Decreased levels of Sirtuin 1 have been shown to be associated with the diagnosis of diabetes mellitus, nonalcoholic fatty liver disease, and the metabolic syndrome, in which epigenetic modifications may play an important role (49, 51–53). Both GGT and HDL-C are metabolic biochemical indicators associated with MetS. The activity of Sirtuin 1 was found to be negatively correlated with GGT levels; Positive regulation of HDL-C levels (54, 55). Thus, inactivation of Sirtuin 1 may also be associated with the optimal threshold GGT/HDL-C ratio for screening MetS. Studies on the relationship between Sirtuin 1 and GGT and HDL-C are limited, and the mechanisms involved are not fully understood. More studies are needed in the future to explore the role of Sirtuin 1 in the relationship between GGT/HDL-C ratio and MetS, which may help to reduce the risk of MetS in subjects with T2DM, to start treatment early, and to improve the quality of life of patients.

The strengths of this study are as follows. (1) We revealed for the first time an independent positive relationship between MetS in subjects with T2DM and GGT/HDL-C ratio; (2) We performed a series of sensitivity analyses (transformation in the form of target independent variables, subgroup analysis) to ensure the reliability of the results. Limitations of this study are also noteworthy: (1) This was a cross-sectional study and therefore only provides weak evidence of an association between exposure and outcome; it is difficult to distinguish a cause-and-effect relationship. Prospective studies are needed to further validate this relationship, especially in comparison with the TyG index. This will help us to understand the validity of the GGT/HDL-C ratio in predicting MetS and its potential application in early detection and intervention. (2) Our study was conducted in Chinese patients with T2DM, and its applicability to other national and ethnic populations is unknown. Additionally, our study was the lack of subpopulation analysis of the number and the kind of metabolic syndrome components. (3) This study lacked data on NAFLD, although we had adjusted for liver enzymes; therefore, the presence of NAFLD could not be considered in the data analysis. (4) As with other observational studies, there may be certain confounding factors that were not identified or included.

## 5 Conclusion

GGT/HDL-C ratio is an independent risk factor for MetS in subjects with T2DM. The risk of MetS in subjects with T2DM increased in conditions with a higher GGT/HDL-C ratio. In the study, GGT/HDL-C ratio was a stronger predictor of MetS than TyG index and HOMA-IR, suggesting as a simple marker for use in underdeveloped and developing countries and in areas with poor laboratory facilities, as well as facilitating large epidemiological studies. For clinical applications, GGT/HDL-C ratios above 19.94 should be used by physicians to guide future medical prophylaxis of MetS in subjects with T2DM to avoid cardiometabolic complications as early as possible.

## Data availability statement

The raw data supporting the conclusions of this article will be made available by the authors, without undue reservation.

## Ethics statement

The studies involving humans were approved by Ethics Committee of Changde First People’s Hospital. The studies were conducted in accordance with the local legislation and institutional requirements. Written informed consent for participation in this study was provided by the participants’ legal guardians/next of kin.

## Author contributions

SGo, SGa analyzed the data and drafted the manuscript. SGa, HZ organized the database. SGo, QZ performed the statistical analysis. HZ wrote sections of the manuscript. All authors contributed to the article and approved the submitted version.

## Glossary

**Table:**

ALT	Alanine aminotransferase
AST	Aspartate transaminase
GGT	Gamma-glutamyl transferase
DBP	Diastolic blood pressure
SBP	Systolic blood pressure
BMI	Body mass index
FPG	Fasting plasma glucose
FIN	Fasting insulin
HbA1c	Glycated hemoglobin
TC	Total cholesterol
TG	Triglycerides
HDL- C	High-density lipoprotein-cholesterol
LDL- C	Low-density lipoprotein-cholesterol
CR	Serum creatinine
UA	Serum uric acid
eGFR	Glomerular filtration rate
TyG index	Triglyceride-glucose index
HOMA-IR	Homeostasis Model Assessment of Insulin Resistance
OR	odd ratio
MetS	Metabolic syndrome
ROC	Receiver-operating-characteristic
AUC	Area under the curve
CI	Confidence interval
T2DM	Type 2 diabetes mellitus
