# Supplementary figures and images for: Gamma-glutamyl transferase to high-density lipoprotein cholesterol ratio is a more powerful marker than TyG index for predicting metabolic syndrome in patients with type 2 diabetes mellitus
Source: Front Endocrinol (Lausanne). 2023 Oct 3;14:1248614. doi: 10.3389/fendo.2023.1248614 (PMC10579940; doi:10.3389/fendo.2023.1248614)

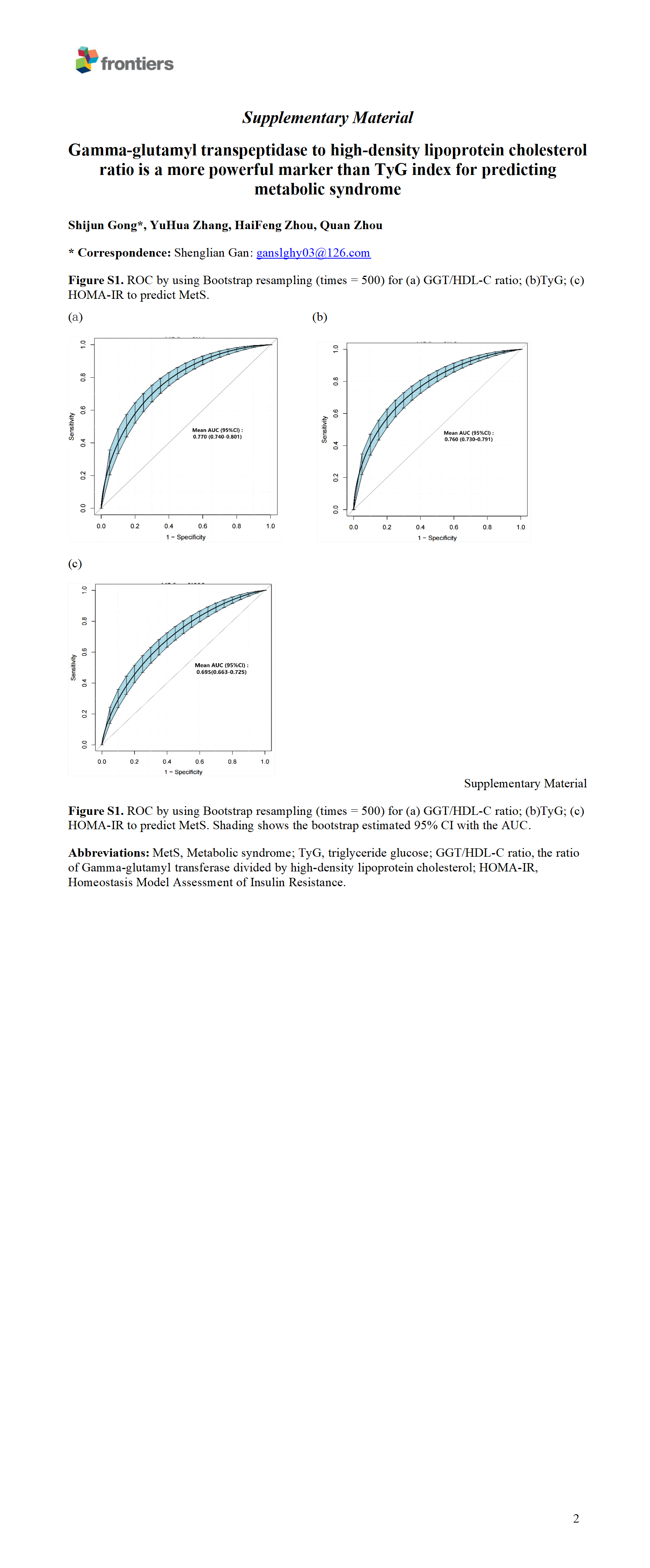

Supplement: Supplementary file 1 [file Image_1.tif]
