# Supplementary material for: Gamma-glutamyl transferase to high-density lipoprotein cholesterol ratio is a more powerful marker than TyG index for predicting metabolic syndrome in patients with type 2 diabetes mellitus
Source: Front Endocrinol (Lausanne). 2023 Oct 3;14:1248614. doi: 10.3389/fendo.2023.1248614 (PMC10579940; doi:10.3389/fendo.2023.1248614)
Supplement: Supplementary file 2 [file Table_1.docx]

Supplementary Material

Gamma-glutamyl transpeptidase to high-density lipoprotein cholesterol ratio is a more powerful marker than TyG index for predicting metabolic syndrome

Shijun Gong*, YuHua Zhang, HaiFeng Zhou, Quan Zhou

*** Correspondence:** Shenglian Gan: [ganslghy03@126.com](mailto:ganslghy03@126.com)

**Supplementary Table 1** The tendency of the prevalence of MetS as the escalation of GGT/HDL-C ratio, TyG index and HOMA-IR

| Variables | Without MetS | With MetS | The prevalence of MetS (%) | P-value |
| --- | --- | --- | --- | --- |
| TyG index |  |  |  | <0.01 |
| ＜1.82 | 214 | 183 | 23.80 |  |
| ≥1.82 | 107 | 586 | 76.20 |  |
| GGT/HDL-C |  |  |  | <0.01 |
| ＜19.94 | 219 | 184 | 23.93 |  |
| ≥19.94 | 102 | 585 | 76.07 |  |
| HOMA-IR |  |  |  | <0.01 |
| ＜3.31 | 206 | 251 | 32.64 |  |
| ≥3.31 | 115 | 518 | 67.36 |  |

**Abbreviations:** MetS, Metabolic syndrome; TyG, triglyceride glucose; GGT/HDL-C, the ratio of Gamma-glutamyl transferas divided by high-density lipoprotein cholesterol; HOMA-IR, Homeostasis Model Assessment of Insulin Resistance.
